# Pathophysiological Links Between Stroke and Prediabetes: A Systematic Review

**DOI:** 10.3390/cimb47100854

**Published:** 2025-10-16

**Authors:** Yerushka Naicker, Andile Khathi

**Affiliations:** Department of Human Physiology, School of Laboratory Medicine and Medical Sciences, College of Health Sciences, University of KwaZulu-Natal, Durban 3629, South Africa; 220014139@stu.ukzn.ac.za

**Keywords:** systematic review, prediabetes, type 2 diabetes mellitus, stroke, stroke risk

## Abstract

Prediabetes is an intermediate stage between normoglycaemia and type 2 diabetes mellitus (T2DM), affecting over 425 million people globally and contributing to vascular damage and increased stroke risk. Despite the severity of both conditions, their association remains underexplored. This review examines the literature on stroke-related biomarkers in normoglycaemia, prediabetes and T2DM to identify potential links between prediabetes and stroke. This systematic review followed PRISMA-2020 guidelines. PubMed, Google Scholar, Scopus, Web of Science and Science Direct were searched for studies (2003–2023) on stroke biomarkers in prediabetes. Eligible studies were original human research in English, with defined diagnostic criteria (ADA or WHO) for glycaemic status and reported biomarker associations or stroke risk. Studies with major comorbidities were excluded. Data were extracted and bias was assessed using the Newcastle–Ottawa Scale. Meta-analysis was not performed due to limited studies per biomarker. Eight studies (*n* = 3003) were included. NSE was examined in three studies, all reporting significant elevations in hyperglycaemic individuals. Interleukin-6 (IL-6) was assessed in two studies; one showed a significant increase in diabetes, while the other found a non-significant upward trend. D-dimer and GFAP were each reported in separate single studies, both showing significant elevations in hyperglycaemic individuals with stroke or neurocognitive impairment. S100B was investigated in two studies, with divergent findings: one showed a positive association with glycaemic status, while the other reported lower levels in hyperglycaemia. Findings indicate biomarker alterations in T2DM, suggesting that early changes may occur in prediabetes. Our review suggests that individuals with prediabetes may show alterations in inflammatory (IL-6), coagulation (D-dimer), and neurovascular (S100B, GFAP, NSE) markers, though some findings are inconsistent, reflecting early pathophysiological changes that may increase stroke risk. Further well-designed studies are needed to clarify these associations and establish biomarker-based tools for earlier stroke risk detection and prevention in individuals with prediabetes.

## 1. Introduction

Type 2 Diabetes Mellitus (T2DM) is a chronic metabolic condition characterised by insulin resistance that results in elevated blood glucose levels [1]. With rapid economic development, dramatic changes in lifestyles and an ageing population, T2DM has become a leading public health problem globally [2]. It is estimated to affect 537 million adults worldwide, with a global prevalence of 10.5% among adults aged 20 to 79 years [3].

Stroke is a well-recognised macrovascular complication associated with T2DM [4]. It is clinically defined as a syndrome of acute, focal neurological deficit attributed to vascular injury, which can either be ischaemic (due to inadequate blood supply) or haemorrhagic (due to bleeding) [5]. Stroke is the second leading cause of death worldwide, accounting for approximately 5.5 million deaths each year [6]. Importantly, individuals with diabetes mellitus and symptomatic cerebral ischaemia have a higher risk of stroke recurrence, and female sex is a demographic factor associated with a worse prognosis [7].

In T2DM, hyperglycaemia leads to oxidative stress, inflammation and endothelial dysfunction, increasing vascular damage and stroke risk [8]. Both T2DM and stroke share common inflammatory pathways; elevated cytokine levels in both conditions accelerate atherosclerosis and enhance thrombogenesis, leading to greater clot formation and ischaemic events [9].

T2DM also impairs cerebral autoregulation and vascular integrity, making the brain more susceptible to ischaemic injury [10]. This can cause reduced cerebral blood flow which contributes to neurodegeneration. Furthermore, insulin resistance affects glucose metabolism in the brain, leaving it more vulnerable to ischaemic events and increasing the risk of stroke [11].

In clinical settings, quick and reliable tests to assess stroke risk and diagnose ongoing stroke events are essential for timely intervention. Current methods, such as CT scans and MRI, although accurate, are not always available in resource-limited settings. Therefore, identifying and using reliable stroke biomarkers is a crucial area of research.

Recent studies have shown that specific biomarkers of inflammation, coagulation and brain injury are effective for evaluating stroke risk and severity in individuals with T2DM [12]. Elevated levels of these biomarkers are linked to higher stroke risk and worse outcomes, indicating their potential role in diagnosis and prognosis.

However, while these associations have been extensively studied in T2DM cohorts, there remains a limited understanding of how such biomarkers change during the earlier stages of dysglycaemia.

Before the onset of T2DM, moderate insulin resistance leads to a gradual increase in glucose levels, known as prediabetes [13]. This condition, also referred to as intermediate hyperglycaemia, is marked by blood glucose levels that are elevated but not high enough to meet the criteria for T2DM [14]. Evidence indicates that complications associated with T2DM often begin during the prediabetes state, with research showing that individuals with prediabetes are at an increased risk of both progression to T2DM and stroke [15].

Yet, no independent synthesis of biomarker data has been undertaken specifically in individuals with prediabetes. Most existing research has inferred stroke risk in prediabetic individuals based on findings from T2DM studies, under the assumption of shared pathophysiological mechanisms. This represents a significant gap in the literature, as it remains unclear whether the same biomarker trends observed in T2DM also apply in earlier dysglycaemic states.

In 2021, the worldwide prevalence of impaired glucose tolerance (IGT) was 9.1%, affecting 464 million people and it is expected to rise to 10.0%, or 638 million individuals, by 2045 [16]. Importantly, prediabetes is increasingly linked to early onset of T2DM-related complications, particularly in low- and middle-income countries where limited awareness and scarce healthcare resources delay diagnosis and management. This contributes to poorer quality of life, increased stroke risk and greater strain on already burdened healthcare systems. Many affected individuals may become unable to work, leading to economic dependency on social grants or family support, which further impacts community and national economies. The rising prevalence of prediabetes among younger populations increases healthcare costs, reduces workforce productivity and elevates morbidity and mortality. Therefore, prioritising early detection, education and prevention is critical to reduce the long-term public health impact of prediabetes and its complications.

Importantly, prediabetes is a reversible condition, and early identification of at-risk individuals could enable interventions to prevent progression to T2DM and reduce stroke risk. This review uniquely synthesises biomarker data reflecting mechanisms underlying both stroke and hyperglycaemia, with a focus on younger adults in populations where prediabetes prevalence is rising. This panel of biomarkers holds promise for independent monitoring of stroke risk in at-risk individuals.

An observational study found that T2DM doubles stroke risk compared to individuals without diabetes [17]. While the association between T2DM and stroke is well-documented, there is a lack of comprehensive research examining stroke in individuals with prediabetes.

There is a need to better understand whether prediabetes itself confers measurable risk in terms of biomarker expression, or if stroke-related pathophysiological changes only emerge at later stages of metabolic dysfunction.

Therefore, this systematic review aims to bridge this gap by examining the pathophysiological mechanisms between prediabetes and stroke in individuals aged 18 and above, addressing the rising prevalence of prediabetes in younger adults and its potential long-term implications for stroke risk. This review will summarise articles that have analysed stroke biomarkers in individuals with type 2 diabetes mellitus and prediabetes.

### 1.1. Research Question

What evidence exists regarding the pathophysiological mechanisms and stroke biomarkers in individuals with prediabetes?

### 1.2. Objectives

Primary objective:

- To summarise the evidence on blood biomarker changes associated with stroke risk in individuals with prediabetes.

Secondary objective:

- To evaluate the reliability and clinical relevance of blood biomarkers for assessing stroke risk and outcomes in individuals with prediabetes.

## 2. Materials and Methods

The protocol for this systematic review has been registered with the International Prospective Registry of Systematic Reviews, PROSPERO; CRD42024530755, as required in the Prepared Reporting Items for Systematic Reviews and Meta-Analysis (PRISMA-P) Protocols 2020 [18].

### 2.1. Eligibility Criteria for the Study

The review targeted prospective observational studies, case–control, cohort and cross-sectional studies that assessed associations of type 2 diabetes, prediabetes and stroke risk through various stroke biomarkers.

This review included only studies written in English. This restriction was due to limited translation resources and the risk of misinterpretation. As most high-impact studies in this field are published in English, the likelihood of capturing relevant evidence remained high. While this may introduce language bias, it ensured the feasibility and accuracy of the review.

Grey literature was not included due to concerns about inconsistent reporting standards and limited peer review, which may compromise the reliability of biomarker data. However, manual screening of reference lists from relevant articles was conducted to help mitigate potential gaps.

All participants with prediabetes or type 2 diabetes aged above 18 years from all ethnic groups were included in this study. Included studies must have had a clear criterion used to diagnose participants with prediabetes or T2DM such as the ADA or WHO criteria. Also, studies that reported participants with a history of stroke or participants who currently experienced a stroke were included.

Studies reporting participants with a history of traumatic brain injury, hypertension, blood disorders, depression and kidney/heart/liver diseases were excluded.

Studies meeting this criterion were considered eligible for the systematic review.

### 2.2. Ethics Approval and Consent to Participate

Data was not collected from individuals; instead, the review analysed previously published data. Since no informed consent was required, the systematic review did not need ethics approval.

### 2.3. Diagnostic Criteria

For this study, eligible participants had to be clinically diagnosed with prediabetes according to the diagnostic criteria set by the ADA or WHO; however, there were some differences in their criteria. For fasting plasma glucose, the ADA defined impaired fasting glucose (IFG) as a level between 5.6 and 6.9 mmol/L, while the WHO specified a range of 6.1 to 6.9 mmol/L. Impaired glucose tolerance (IGT) was defined as a 2-h plasma glucose measurement. Both ADA and WHO used the same criteria for IGT, setting the range at 7.8 to 11.0 mmol/L. Unlike the WHO, the ADA included an HbA1c guideline for assessing glucose levels, specifying a range of 5.7 to 6.4%. Despite the minor differences, both the ADA and WHO criteria provided clear guidelines designed to diagnose prediabetes, as depicted in Table 1. Only studies that used the ADA/WHO criteria were included. Since different countries use different diagnostic standards, including both criteria allowed us to capture the largest number of relevant studies while maintaining clinical accuracy.

In this review, stroke diagnosis was primarily based on clinical criteria as reported in the respective studies, including neurological assessments and imaging techniques such as computed tomography (CT) scans and magnetic resonance imaging (MRI), where available. However, if the diagnostic method for stroke was not explicitly stated, we included studies that met the broader clinical definitions according to the World Health Organization (WHO) or American Heart Association (AHA) guidelines, which define stroke as a sudden onset of neurological symptoms caused by an interruption of blood flow to the brain.

### 2.4. Search Strategy

The databases screened were PubMed, Google Scholar, Scopus, Web of Science and Science Direct. The Boolean Logical operator “AND” and “OR” was used between search terms. Additional filters were applied to narrow results. To refine the results, additional filters were applied: studies involving humans aged 18 and older, published between 2003 and 2023 and available as free full text. This timeframe was chosen to include the most recent research and avoid outdated information. Some of the following medical subject headings were used in our search strategy: “prediabetes AND stroke risk,” “type 2 diabetes mellitus AND stroke” AND “NSE OR D-dimer OR GFAP OR IL-6”. Additionally, bibliographies of relevant articles were manually screened for other relevant articles. A comprehensive PubMed search strategy is provided in Table 2.

### 2.5. Identification of Eligible Studies

**Figure 1:**
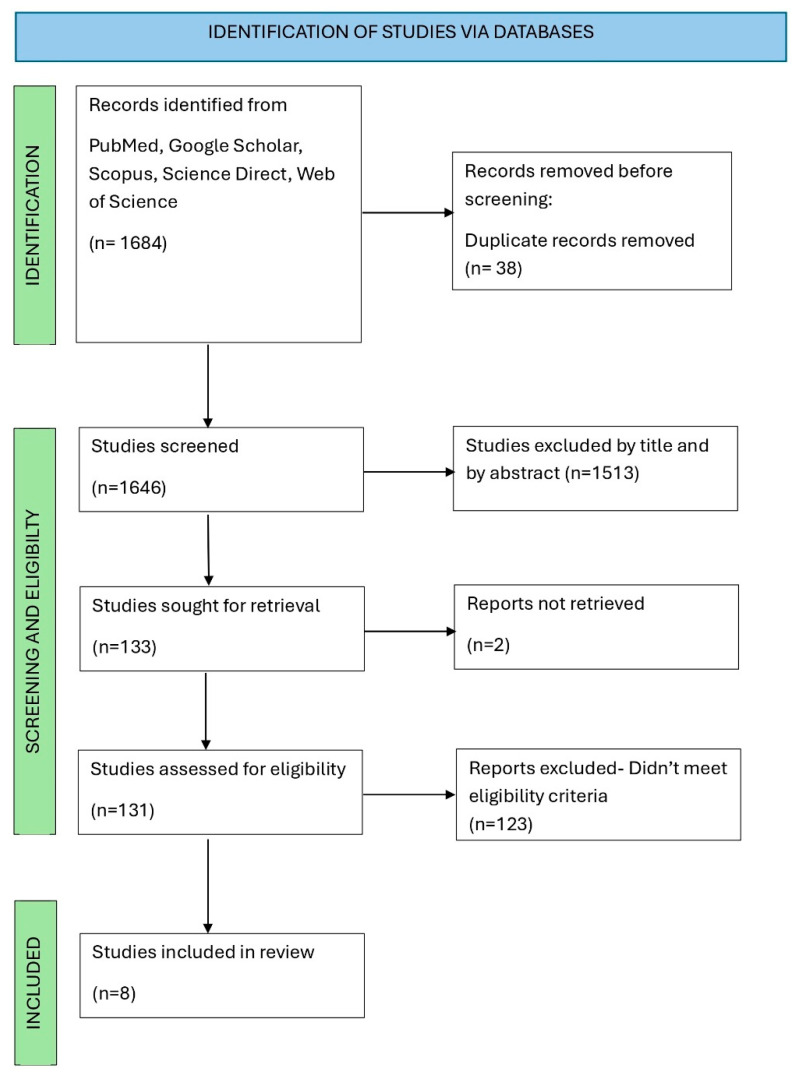
Preferred reporting items for systematic review flow diagram of included studies.

Duplicate studies were removed using Endnote X20 reference manager. YN screened the articles and removed irrelevant studies based on the titles and abstracts. Studies were deemed eligible and included in the review if they met the stated inclusion criteria. Thereafter, YN conducted a second screening of all articles to ensure accuracy and consistency. Uncertainty of results was settled by AK. The selection process is represented in the PRISMA flow chart (Figure 1).

### 2.6. Data Extraction

Relevant data were extracted from the selected studies and recorded in a Microsoft Excel file. Among the baseline characteristics extracted from the eligible reports included author, publication year, country, study setting, methodology categorised into study design, number and age range of participants. Quantitative data, including biomarker levels and statistical significance, were extracted and organised for comparative analysis.

In addition to biomarkers, qualitative information regarding the pathophysiological mechanisms explaining the link between prediabetes or type 2 diabetes mellitus and stroke was also extracted. Proposed mechanisms, such as insulin resistance, chronic inflammation, endothelial dysfunction and vascular damage induced by hyperglycaemia, are described in detail. Theoretical discussions had been summarised and then categorised into distinct mechanisms to allow a comprehensive understanding of how these processes contribute to the risk of stroke in individuals with prediabetes. Accordingly, these pathophysiological insights have been synthesised and reported in the narrative sections of this review, complementing the quantitative data on biomarkers of stroke.

### 2.7. Risk of Bias and Quality Appraisal

The potential risk of bias was assessed by YN using a risk of bias checklist. The Newcastle Ottawa quality assessment scale checklist was used as it assessed the quality of cohort, cross-sectional and case–control studies (newcastle—ottawa quality assessment scale. (n.d.). Newcastle—ottawa quality assessment scale. https://cdn-links.lww.com/permalink/eandh/a/eandh_1_1_2021_02_03_meghji_eandh-d-20-00242_sdc3.pdf) (accessed on 15 October 2024). Risk of bias was assessed for all 8 studies included in the final review. Given the limited number of eligible studies, we retained all eight, regardless of their NOS scores. However, studies with lower methodological quality were interpreted with caution during synthesis and their findings were weighed accordingly. This approach allowed for a comprehensive review while maintaining transparency and critical appraisal standards. Disagreements were settled by an independent researcher. The overall quality of the included studies was good with a score of 8/9.

### 2.8. Data Synthesis and Analysis

While forest plots were initially planned for statistical aggregation, the limited number of studies available for each marker made this approach unfeasible. As a result, the findings were presented without the usual meta-analysis aggregation. Results from the included studies were first collated into a summary table and then synthesised narratively. Studies were grouped according to shared biomarker targets and synthesised based on their associated pathophysiological mechanisms, such as inflammation, endothelial dysfunction and neuronal injury. This thematic organisation allowed for comparison of biomarker trends across populations with T2DM and prediabetes, enabling the identification of patterns and gaps relevant to stroke risk.

## 3. Results

### 3.1. Search Report Results and Eligible Reports

The search strategy generated 1684 studies from the following databases: PubMed, Google Scholar, Scopus, Science Direct and Web of Science. A total of 38 duplicates were removed before screening, and 1513 studies were excluded based on review of the title and abstracts. 133 studies were sought for retrieval, of which only 131 were assessed for eligibility. Thereafter, 123 studies were excluded after full text analysis as they did not meet the eligibility criteria or report on stroke biomarkers in groups of individuals with T2DM or prediabetes. Hence, a total of 8 studies were included in this systematic review.

### 3.2. Study Characteristics

All included studies were of those published in journals between 2003 and 2023. The total number of participants across the included eight studies is 3003. Study characteristics and results have been summarised and presented in Table 3. The results have been grouped according to the investigated pathophysiological mechanisms, namely inflammation, coagulation, neurovascular injury and further categorised by specific biomarker type to facilitate clearer interpretation of patterns, consistencies and contradictions across studies.

## 4. Discussion

The global prevalence of prediabetes is significant and, on the rise, particularly in low-income countries, where the most substantial growth is anticipated by 2045. In 2021, the global prevalence was 9.1%, affecting 464 million people, and this is expected to rise to 10.0%, impacting 638 million by 2045 [16]. Additionally, IGT prevalence is projected to grow in adults aged 20–74 years, emphasising the need for intervention [16].

Individuals with prediabetes are at a higher risk of developing T2DM and its associated complications [19]. An investigation conducted in 2019 found that, in comparison to people with normal glucose levels, those with prediabetes had a higher risk of having a new stroke and a worse outcome [20].

While evidence is available that links prediabetes with an increased risk of stroke, the mechanisms through which this happens are not well-defined. Further challenges remain in explaining how elevated blood glucose levels during the prediabetes state may facilitate the development of cerebrovascular disease. Even though the diagnosis of stroke today is optimally performed through neuroimaging studies using CTs or MRIs [21], there is still a dire need for quicker and easier diagnostic tools, especially in resource-deprived settings.

Studies have shown that certain blood biomarkers may be a reliable predictor in assessing the risk of stroke or facilitating its diagnosis [22,23,24]. With the rising global incidence of stroke and prediabetes, especially among the younger population, this is a concern that should be given even greater emphasis. This systematic review explored the association between prediabetes and stroke through significant biomarkers like IL-6, D-dimer, NSE and GFAP. Further, this review also tried to elucidate the underlying pathophysiological mechanisms in the state of prediabetes that increase susceptibility to stroke or trigger stroke events.

It was, however, hard to determine studies suitable for this review since few studies have been conducted concerning this subject. To maximise inclusivity, we applied both the ADA and WHO criteria for prediabetes. We anticipated that using these broader international criteria would allow us to capture more studies; however, despite this approach, the retrieved studies still did not specifically investigate individuals with prediabetes. Although prediabetes is a prevalent condition worldwide, it often goes undetected due to its asymptomatic nature, hence contributing to the deficiency of research concerning this topic.

This study aimed to summarise the existing evidence on the pathophysiological mechanisms linking stroke and stroke biomarkers in individuals with prediabetes. However, despite a thorough search, no studies were found that directly investigated this specific relationship. Instead, this study identified numerous articles assessing various stroke biomarkers in individuals who had experienced a previous stroke, though these did not specifically address prediabetes as a potential predisposing factor. Several researchers also explored the diagnostic value of certain stroke biomarkers in individuals with established T2DM.

While this focus on T2DM was not the original target of the study, the findings remain highly relevant. Since prediabetes is well established as an intermediary state on the glycaemic spectrum, sharing key metabolic disturbances with T2DM, including insulin resistance, low-grade inflammation and endothelial dysfunction, it is biologically plausible that the same biomarkers elevated in T2DM-associated stroke may also exhibit early alterations in individuals with prediabetes, though likely to a lesser degree.

These early molecular changes may indicate subclinical neurovascular injury or coagulation imbalances that precede overt cerebrovascular events. This supports the concept of a continuum of vascular risk, positioning prediabetes as a critical window for early detection and intervention through sensitive biomarker monitoring—even before structural or clinical signs of stroke emerge.

### Pathophysiology Mechanisms Linking Stroke and Type 2 Diabetes Mellitus

In T2DM, both insulin resistance and hyperglycaemia are important components that predispose individuals to cerebrovascular events such as stroke [9]. Insulin resistance results in a hyperglycaemic state due to the disruption of the normal glucose metabolism within the body [25]. Such disruptions cause a cascade of pathological changes that will eventually compromise vascular health.

Inflammation is a basic pathophysiological process that highly contributes to the development of stroke and increased stroke risk [26]. IL-6 is an important pro-inflammatory cytokine, very often present in higher levels in individuals with T2DM and prediabetes [27]. Chronic elevation of IL-6 is associated with enhanced inflammation and endothelial dysfunction, a condition wherein the endothelial cells lining the blood vessels become impaired [27]. These cells generally regulate the flow of blood through the vessel and maintain vascular integrity. However, in a state of chronic inflammation resulting from high levels of IL-6, there is decreased production of nitric oxide, a molecule important for the dilating of blood vessels [28]. Thus, the blood vessels have a reduced capacity to dilate properly, leading to increased stiffness and heightened susceptibility to injury.

Findings from two eligible studies in this systematic review portray conflicting perspectives regarding the role of IL-6 in type 2 diabetes and its complications. The study by [29] reports that high levels of IL-6 are greatly associated with the development of macrovascular complications among individuals with T2DM, where 54.9% of the subjects developed such complications. IL-6 concentrations were significantly elevated in individuals with diabetes and atherosclerosis (1.7 ± 0.5 ng/mL) compared to those without, supporting its role as a pro-inflammatory marker in vascular damage.

This means that the development of atherosclerosis in individuals with diabetes is contributed to by inflammatory factors, including IL-6. The study by [30] found that plasma IL-6 levels in individuals with diabetes were significantly higher than those in individuals without diabetes. However, it also found that IL-6 levels were paradoxically lower in diabetic individuals with complications (0.9 pg/mL) compared to those without complications (7.2 pg/mL), suggesting variability in IL-6 expression depending on disease stage or immune adaptation. It was also noted that there was no significant difference in the level of IL-6 between the individuals with diabetes and the group of individuals with diabetes with microvascular or macrovascular problems.

These findings together emphasise the complexity of inflammation in T2DM. Reference [28] suggests that there is a direct association between raised IL-6 and stroke risk, whereas [30] suggests that other inflammatory pathways may be more important. This strengthens the need for further research to better understand these relationships by having the group of individuals with prediabetes as a testing group in future studies. Understanding these mechanisms in earlier metabolic stages may help determine whether IL-6 elevations precede vascular complications or simply reflect existing pathology.

Interestingly, IL-6 also promotes insulin resistance, where further worsening of hyperglycaemia exacerbates vascular health [31]. This would then form a vicious circle, promoting increased stroke risk in individuals with T2DM.

In this situation, platelet aggregation is one of the very important risk factors. It is so easily regulated by increased IL-6 that it supports platelet activation and fast aggregation of platelets. This might lead to blood clotting, which in turn would cause blockage of blood, thereby increasing the risk of stroke events. Given that prediabetes is marked by low-grade inflammation and early endothelial dysfunction, IL-6 may serve as a marker for detection through sensitive biomarker monitoring in this group, offering a window of opportunity for early intervention before overt macrovascular damage occurs.

Another important biomarker in the assessment of stroke risk is D-dimer. The high level of D-dimer, just like the case of platelet aggregation, expresses increased coagulability, which can lead to thrombus formation and subsequent strokes [32]. A 4-year cohort study in China has associated high levels of D-dimer in individuals with diabetes with increased incidence of cardiovascular events, including ischaemic stroke events, hence the importance of monitoring this biomarker in the prediabetes setting [33]. Although D-dimer was assessed in only a single included study, the large-scale nature of this investigation considerably strengthens the reliability of its findings. This was the only study among the reviewed literature to examine such a large population (*n* = 1976), significantly enhancing its statistical power relative to other biomarker studies. The study by [33] demonstrated a strong and statistically significant association between elevated D-dimer concentrations and increased risk of cardiovascular events, including ischaemic stroke, in individuals with diabetes.

Despite the strength of its design and low risk of bias, the lack of replication across multiple independent studies limits the broader generalisability of these findings. Further research is needed to validate the diagnostic and prognostic utility of D-dimer, especially in prediabetic populations or in studies that specifically stratify stroke subtypes.

When stratified by D-dimer quartiles, the incidence of cardiovascular events rose progressively with *p* < 0.001 for all comparisons. This dose–response trend highlights a strong and statistically significant link between D-dimer concentrations and vascular risk.

This evidence aligns with broader research indicating that hyperglycaemia exacerbates both inflammatory and prothrombotic pathways, thereby increasing the likelihood of macrovascular complications such as stroke. Consequently, these findings may be extended to help explain intermediary pathophysiological mechanisms observed in individuals with prediabetes. This convergence of evidence across distinct pathophysiological pathways highlights D-dimer’s potential role as a complementary marker in multi-biomarker panels aimed at early identification of stroke risk in metabolic disease contexts.

GFAP is an important marker for brain damage in stroke diagnosis. GFAP is released into the bloodstream following brain cell death or injury [34]. GFAP levels can be used as an indicator of the extent of brain damage and can also be used to distinguish between stroke types and a stroke from other causes [35].

This systematic review revealed only one study that examined the relationship between GFAP, cognitive impairments and type 2 diabetes mellitus. A case–control/cohort study by Guerro et al. reported that GFAP levels were considerably higher in patients with neurocognitive disorders compared to healthy controls and the highest concentrations were found in individuals with both neurocognitive disorders and type 2 diabetes [36]. This is concerning since elevated GFAP levels are associated with central nervous system damage and stroke risk is increased by type 2 diabetes, which further exacerbates the problem. Increased GFAP is a sign of an astrocyte’s response to brain damage, which is made worse by type 2 diabetes. This neuroinflammation and dysfunction most likely increases the risk of stroke even more. Although GFAP was identified as a potentially useful biomarker, it was assessed in only a single included study, limiting the generalisability and strength of the conclusions. The study by [36] was assessed as low risk of bias, with clearly reported quantitative findings and appropriate adjustment for confounding variables. However, the lack of replication across multiple independent studies highlights the need for further research to validate GFAP’s diagnostic and prognostic utility in diabetic populations at risk for stroke or neurovascular complications. This trend mirrors that of D-dimer, where consistent associations with stroke risk have been observed, yet limited study replication in prediabetic or diabetic cohorts hinders broader clinical application. Despite this limitation, the consistent elevation of GFAP in T2DM-related neurocognitive and vascular conditions lends support to its potential role as an early biomarker of astrocytic dysfunction and warrants future exploration in larger, well-controlled studies.

Following brain injury, damaged astrocytes release the calcium binding protein S-100B, which is mostly located in the central nervous system [37]. Serum levels can be indicative of several pathological conditions, such as stroke and diabetes. Following an ischaemic stroke, S100B has been reported to be released into the bloodstream as a result of glial cell activation and breakdown of the blood–brain barrier (BBB) [38].

Another example is Neuron-Specific Enolase (NSE), a biomarker of neuronal injury. NSE is usually elevated in diabetes and this could be related to greater neuronal injury and poorer prognosis in individuals with diabetes who may have experienced a stroke event [39]. Among all biomarkers reviewed, NSE demonstrated the strongest and most consistent evidence as a marker of neurovascular injury. Three independent studies, encompassing a combined sample size of 361 participants, consistently reported statistically significant elevations in NSE levels both in individuals with diabetes compared to healthy controls, and in individuals with diabetes who experienced ischemic stroke compared to diabetic individuals without stroke. This reproducibility across diverse populations strengthens confidence in NSE’s reliability as a biomarker of neurovascular injury.

Regarding study quality, two of the studies [40,41] were assessed as having low risk of bias and provided detailed quantitative data with clear statistical comparisons, enhancing the interpretability and validity of their findings. In contrast, one study [42] was rated as high risk of bias due to reliance on self-reported outcomes via telephonic follow-up, a small sample size and lack of randomization. Moreover, this study reported only statistically significant differences (*p* < 0.05) between diabetic and non-diabetic acute ischemic stroke patients without providing specific NSE concentration values, limiting comparability with other studies.

Despite this limitation, the overall consistency of elevated NSE levels in diabetic stroke patients across the low-bias studies supports NSE’s clinical utility as a reliable biomarker for neurovascular injury. Its consistent elevation in diabetic stroke groups versus diabetic controls indicates NSE’s potential to discriminate neurovascular injury severity within diabetic populations.

Similarly to S-100B, the levels of NSE are accepted as a marker of the severity of brain damage in stroke patients, including those with diabetes, since the damaged BBB allows NSE to leak out into the bloodstream.

Roshdy et al. found that elevated levels of S100B and NSE correlated significantly with infarct size and neurological examinations, indicating their diagnostic validity [40]. Additionally, the study by Pandey et al. highlighted the use of NSE as a reliable prognostic marker of stroke severity and outcome in individuals with elevated blood glucose levels [41].

Interestingly, the study by [43] found that in T2DM, S100B is implicated in cognitive dysfunction, a common complication among patients. The relationship between S100B levels and cognitive impairment suggests that this biomarker may reflect both acute neuronal injury and chronic neurodegenerative processes linked to diabetes. Additionally, the study revealed an association between chronic hyperglycaemia and cognitive function, reporting lower S100B levels in individuals with T2DM compared to healthy controls. This reduction is thought to be due to hyperglycaemia downregulating S100B secretion from astrocytes. However, the findings also indicate that lower S100B levels are associated with worse cognitive function, suggesting that while insulin resistance may lead to reduced S100B, the protein could still play a critical role in neuroprotection and cognitive health.

Interestingly, although the included studies primarily focused on individuals with T2DM and stroke, regardless of the biomarker investigated, most participants were middle-aged or older, typically between 50 and 60 years of age. This highlights a gap in current research, as evidence shows that prediabetes often develops at a younger age. In addition, individuals over 50 years are more likely to have other comorbidities, which can complicate the interpretation of biomarker changes and stroke risk. Therefore, future studies should consider focusing on younger populations, particularly those aged 25 to 45 years, to better isolate the early pathophysiological effects of prediabetes and gain clearer insights into its association with stroke risk.

The exact role of biomarkers S100B, GFAP and NSE in diagnosing stroke is still being defined. While elevated levels of these markers indicate neuronal injury, they are not unique to stroke and can also be present in other neurological conditions [44,45].

The results of this systematic review, along with findings from existing literature, indicate that S100B, GFAP and NSE hold promise as biomarkers for brain injury in stroke, particularly in individuals with T2DM. By combining these markers with others that evaluate different pathophysiological pathways associated with stroke such as IL-6 and D-dimer, we can develop a comprehensive panel of blood biomarkers that may provide a more accurate clinical prognosis for stroke patients with prediabetes and T2DM. Although most included studies focused on individuals with established T2DM, the underlying pathophysiological mechanisms are highly relevant to prediabetes. Insulin resistance, a hallmark of prediabetes, contributes to chronic low-grade inflammation and endothelial dysfunction, which can in turn influence levels of circulating biomarkers such as IL-6, D-dimer, and S100B. Given the continuum of metabolic dysfunction between normoglycaemia and overt T2DM, it is biologically plausible to expect intermediate or subclinical elevations in these biomarkers among individuals with prediabetes. Such elevations may reflect early neurovascular stress or low-grade neuroinflammation that precedes overt clinical stroke, offering a critical window for early intervention. This highlights the importance of exploring biomarker trajectories in prediabetes, not only to better understand the early pathogenesis of stroke, but also to identify at-risk individuals before irreversible damage occurs. Future studies should focus on investigating these biomarkers specifically in individuals with prediabetes and T2DM to further clarify their diagnostic value and enhance clinical outcomes.

## 5. Limitations

The main limitation of this study was the lack of data on changes in stroke biomarker concentrations in individuals with prediabetes. All 8 studies included in this review assessed various stroke biomarkers in individuals with T2DM. Since prediabetes is an asymptomatic condition, many individuals are unaware that they have it. For individuals who suffer a stroke event but were not diagnosed with prediabetes, prediabetes may not be considered a possible cause of the stroke. These individuals have not been included in studies that investigate the relationship between diabetes and stroke. Additionally, factors like gender and race were not always specified in studies. While we know that prediabetes and stroke are becoming more prevalent in younger adults, most studies only investigated in individuals over the age of 50.

## 6. Conclusions

It is well-established that T2DM increases the risk of stroke due to mechanisms such as insulin resistance, chronic inflammation, endothelial dysfunction and hyperglycaemia-induced vascular damage. Several studies have confirmed these mechanisms and investigated stroke biomarkers, such as IL-6, S100B, NSE, D-Dimer and GFAP in T2DM populations, demonstrating their relevance to stroke risk. While some articles have also explored these mechanisms in individuals with prediabetes, providing evidence of an elevated stroke risk, there remains a significant gap in research focusing specifically on stroke biomarkers in populations with prediabetes. Further studies are needed to better understand the biomarker profiles associated with stroke in prediabetes, which could help identify early interventions to reduce stroke risk in this group.

## Figures and Tables

**Table 1:** Difference between the American Diabetes Association and World Health Organization criteria for prediabetes diagnosis.

Criteria	ADA	WHO
Fasting Plasma glucose (IFG)	5.6–6.9 mmol/L	6.1–6.9 mmol/L
2 Hour plasma glucose (IGT)	7.8–11.0 mmol/L	7.8–11.0 mmol/L
HbA1c %	5.7–6.4%	-

**Table 2:** PubMed search strategy for studies.

Search	Search Terms
1	Search (“Prediabetes”[MeSH Terms] OR “Impaired Glucose Tolerance” OR “Type 2 Diabetes Mellitus”)
2	Search (“Stroke”[MeSH Terms] OR “Stroke Risk” OR “Cerebrovascular Accident”)
3	Search (“NSE” OR “Neuron-Specific Enolase” OR “D-dimer” OR “GFAP” OR “Glial Fibrillary Acidic Protein” OR “IL-6” OR “Interleukin-6” OR “S100B” OR “Fibrinogen” OR “Stroke Biomarkers”)
4 (Additional Filters)	Humans, Age 18+, English, Free Full Text, Publication Date: 2003–2023
5	Search (#1 AND #2 AND #3 AND #4)

**Table 3:** Overview of characteristics of the included studies.

Biomarker	Author (Year)	Study Design	Country	Population (n)	Avg. Age	Diabetes Status	Stroke Type/Condition	Key Findings	Risk of Bias
Neurovascular Injury									
NSE	Pandey et al. (2011)	Cross-sectional case–control	India	191	60.5	Diabetes Mellitus	Ischaemic Stroke	Stroke: 18.0 ± 4.5 ng/mL Controls: 7.5 ± 1.5 ng/mL (*p* = 0.001) Normoglycemic Ischaemic Stroke Patients: 15.2 ± 2.4 ng/mL Hyperglycaemic Ischaemic Stroke Patients: 19.7 ± 4.7 ng/mL (*p* = 0.05).	Low
NSE	Nayak et al. (2016)	Cohort	India	104	50	Type 2 Diabetes Mellitus	Ischaemic Stroke	NSE < 0.65 µg/L in controls. Significant group differences but specific *p*-values not given.	High
NSE	Roshdy et al. (2007)	Cross-sectional	Egypt	66	52.3	Type 2 Diabetes Mellitus	Ischaemic Stroke	Diabetic Stroke: 14.42 ± 4.09 µg/L Controls: 6.71 ± 1.29 µg/L (*p* < 0.001) Strong positive correlation with infarct size (r = 0.938).	Low
S100B	Roshdy et al. (2007)	Cross-sectional	Egypt	66	52.3	Type 2 Diabetes Mellitus	Ischaemic Stroke	Diabetic Stroke: 115.18 ± 7.45 µg/L vs. Controls: 28.22 ± 7.9 µg/L (*p* < 0.001) Strong correlation with infarct size (r = 0.9816).	Low
S100B	Yu et al. (2020)	Case–control	Turkey	164	49.67	Type 2 Diabetes Mellitus	Cognitive Dysfunction	Lower levels in T2DM with cognitive dysfunction (0.117 µg/L) vs. controls (0.344 µg/L) (*p* < 0.05).	Moderate
GFAP	Ayala-Guerrero et al. (2022)	Comparative study	Mexico	138	72.3	Type 2 Diabetes Mellitus	Neurocognitive Disorders	GFAP higher in NCD with T2D (2046 pg/mL) vs. control without T2D (583.2 pg/mL) (*p* < 0.0001).	Moderate
Inflammation									
IL-6	Pickup et al. (2000)	Case–control	UK	37	55.4	Type 2 Diabetes	Diabetic Complications	T2D: 1.8 pg/mL vs. Controls: 1.1 pg/mL (*p* < 0.01); No difference between diabetics with and without complications.	Low
IL-6	Wu et al. (2012)	Case–control	China	327	55	Type 2 Diabetes Mellitus	Atherosclerosis	Higher IL-6 in both diabetic and non-diabetic groups with atherosclerosis vs. without (*p* = 0.007 and *p* = 0.004, respectively).	Low
Coagulation									
D-Dimer	Cheng et al. (2022)	Retrospective cohort	China	1976	59.6	Type 2 Diabetes Mellitus	Stroke Events (general)	Higher quartiles of D-dimer levels associated with more stroke events. *p* < 0.001 across all quartile comparisons.	Low

## Data Availability

The raw data supporting the conclusions of this article will be made available by the authors on request.

## Abbreviations

The following abbreviations are used in this manuscript:

ADA	American Diabetes Association
AHA	American Heart Association
BBB	Blood–brain barrier
CT	Computed tomography
GFAP	Glial fibrillary acidic protein
HbA1c	Glycated Haemoglobin
IFG	Impaired fasting glucose
IGT	Impaired glucose tolerance
IL-6	Interleukin 6
MRI	Magnetic resonance imaging
NSE	Neuron-specific enolase
PRISMA	Prepared Reporting Items for Systematic Reviews and Meta-Analyses
S100B	Calcium-binding protein B
T2DM	Type 2 diabetes mellitus
UKZN	University of KwaZulu-Natal
WHO	World Health Organization
